# Barcoding Populations of *Pseudomonas fluorescens* SBW25

**DOI:** 10.1007/s00239-023-10103-6

**Published:** 2023-04-25

**Authors:** Loukas Theodosiou, Andrew D. Farr, Paul B. Rainey

**Affiliations:** 1 Department of Microbial Population Biology, Max Planck Institute for Evolutionary Biology, Plön, Germany; 2 Department of Comparative Development and Genetics, Max Planck Institute for Plant Breeding, Cologne, Germany; 3 Laboratory of Biophysics and Evolution, CBI, ESPCI Paris, Université PSL, CNRS, Paris, France

## Abstract

In recent years, evolutionary biologists have developed an increasing interest in the use of barcoding strategies to study eco-evolutionary dynamics of lineages within evolving populations and communities. Although barcoded populations can deliver unprecedented insight into evolutionary change, barcoding microbes presents specific technical challenges. Here, strategies are described for barcoding populations of the model bacterium *Pseudomonas fluorescens* SBW25, including the design and cloning of barcoded regions, preparation of libraries for amplicon sequencing, and quantification of resulting barcoded lineages. In so doing, we hope to aid the design and implementation of barcoding methodologies in a broad range of model and non-model organisms.

**Supplementary Information:**

The online version contains supplementary material available at 10.1007/s00239-023-10103-6.

## Introduction

Barcoding individual microbial cells with chromosomally integrated short, random sequences is a powerful approach for obtaining a high-resolution understanding of the eco-evolutionary dynamics that shape microbial populations (Blundell and Levy 2014; Levy et al. 2015; Cvijović et al. 2018; Ba et al. 2019; Kinsler et al. 2022; Johnson et al. 2023). In brief, the technique involves introducing a large number of random sequences into a single chromosomal region of a population of cells such that all lineages are uniquely identifiable. A single PCR reaction allows amplification of the barcoded region, while DNA sequencing of the product reveals the frequency of each unique code. Changes in barcode frequencies allow inference of mutational events and fitness effects. For example, a cell that acquires a beneficial mutation will cause the frequency of the linked barcode to increase within the population. The rate of increase can be used to quantitate contributions to fitness.

When changes are measured over the course of hundreds of generations, detailed understanding of eco-evolutionary dynamics can emerge (Levy et al. 2015; Jasinska et al. 2020; Acar et al. 2020; Boyer et al. 2021; Aggeli et al. 2021), including insight into the connection between genotype and phenotype of even complex traits (Venkataram et al. 2016; Kinsler et al. 2020; Jagdish and Nguyen Ba 2022; Nguyen Ba et al. 2022; Aggeli et al. 2022; Ascensao et al. 2022). Recent developments allowing the regeneration of barcode diversity extend the opportunity to observe evolutionary change over the course of thousands of generations (Ba et al. 2019).

While application of barcoding initially focussed on adaptive evolution in single populations of model microbes, particularly yeast (*Saccharomyces cerevisiae*) (Blundell and Levy 2014; Cvijović et al. 2018) and *Escherichia coli* (Jasinska et al. 2020), strategies have since been devised for studying the evolution of cancer using barcoded lung cancer cells in mice (Rogers et al. 2018) and quantification of eco-evolutionary dynamics in microbial communities (Venkataram et al. 2022). The potential for extending strategies and applications is considerable, ranging from tracing object provenance (Nguyen Ba et al. 2022), enhancing experimental power through mutant-multiplexing (Jackson et al. 2020), extending understanding of microbiome transmission (Vasquez et al. 2021), to the study of demographic changes during colonisation of new environments (Brettner et al. 2022). However, one impediment to progress is the effort required to develop the initial barcoded library, especially in non-model systems.

Here, we outline a step-by-step guide for barcoding microbial cells, with a particular focus on the bacterium *Pseudomonas fluorescens* SBW25 (hereafter SBW25). In addition to detailed protocols covering library preparation, amplicon-sequencing methods and strategies for assessment of data quality, critical steps are emphasised and guidance of a general nature given in order to facilitate the development of barcoded libraries in non-model microbes.

## Library Creation and Validation

### Design and Construction of Barcoded Integrative Plasmids

The primary aim is to introduce barcoded sequences into the chromosome of an otherwise isogenic population in order to enable lineage tracking in later experiments. Note that barcoding for lineage tracking is distinct from ‘Tn-seq’ (van Opijnen et al. 2009), which introduces transposon insertion mutations along with a barcode sequence. The standard strategy behind barcoding for lineage tracking involves the creation of a vector with a region of extreme sequence variation, which is eventually introduced into the target bacterium. The backbone vector should have minimal fitness consequences and should integrate just once at a single genomic site. In addition, the ‘barcode’ region should be short enough to be sequenced with short-read next-generation sequencing platforms. The downstream library of barcoded mutants should also be highly diverse (i.e. there should be many barcodes, each at a similar frequency).

For our libraries, derivatives of mini-Tn*7* vectors (Choi and Schweizer 2006) were employed. This transposon-based system ensures chromosomal integration of random (barcode) sequences in a single, known chromosomal location (Waddell and Craig 1989). The strategy is similar to that used to barcode *E. coli* (Jasinska et al. 2020). The advantage of the Tn*7* system is the highly conserved position of the *att*Tn*7* site (downstream of the *glmS* gene) into which Tn*7* elements integrate (without causing the inactivation of an open-reading frame). In addition, the *att*Tn*7* region is conserved across many bacterial phyla, including Proteobacteria, Fermicutes, and Bacteroidetes (Parks and Peters 2007; Mitra et al. 2010; Wiles et al. 2018), and integration of Tn*7*-vector systems has been demonstrated across many model organisms (Schlechter et al. 2018; Wiles et al. 2018), including SBW25 (Liu et al. 2014; Lind et al. 2015; Farr et al. 2017). In bacteria lacking an *att*Tn*7* site, such a site can be engineered into the genome, provided that the bacterium of interest is capable of transformation and undergoing allelic replacement (Figueroa-Cuilan et al. 2016). If introduction of *att*Tn*7* is not an option, other methods are available for introducing barcodes, but they are less ideal for final applications. These options include (1) the transformation of cells with barcoded plasmids (Vasquez et al. 2021) and (2) the use of Tn*5*-based systems. The former requires selection for maintenance of the plasmid during subsequent evolution experiments, and this can be problematic from the perspective of cost, and variation in copy number complicates estimates of barcode lineage frequency. The Tn*5*-based option involves use of transposons that integrate effectively at random with each insertion standing to disrupt targeted genes. This is clearly undesirable.

**Fig. 1:**
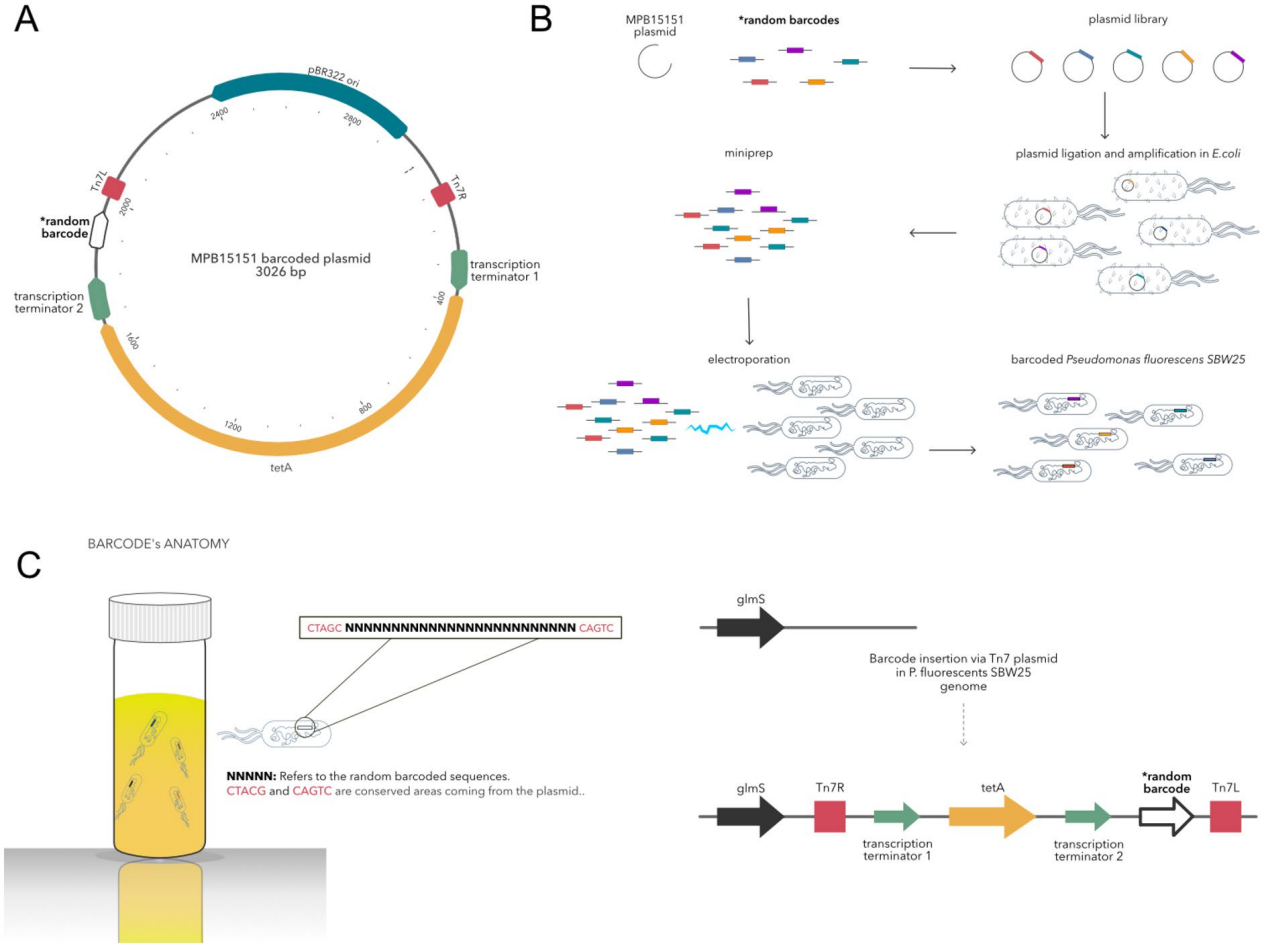
Barcoding *Pseudomonas fluorescens* SBW25 cells. Panel A shows a genetic map of the ~ 3 kb vector used to introduce barcoded sequences into the chromosome of SBW25. The vector features an origin of replication (pBR322) which allows replication solely in *E. coli* hosts, but not *Pseudomonas*, two Tn*7* integration elements that enable integration of intermediate DNA at the *att*Tn*7* site, a gene conferring tetracycline resistance (*tetA*) (allows selection for transformants), two transcription regulators to limit unwanted transcription of *tetA* and the barcode, and finally the barcoded region, consisting of 25 random base pairs.

In developing an integration vector for SBW25, all efforts were made to keep the vector as small as possible and the metabolic cost of the vector as minimal as possible to prevent selection for lineages with spontaneous deletions of the barcoded region in subsequent experiments. The final vector is ~ 3000 bp and contains a minimal set of expressed genes (Fig. 1, Panel A). Limiting the size of the vector has the additional benefit of increasing the rate of transformation (although it should be noted that a co-transformation with a larger helper vector is required for the integration of mini-Tn*7*-vectors). Our vector was constructed using Gibson assembly, although any assembly method can be used, including standard restriction enzyme digestion and ligation strategies.

Panel B summarises the molecular process required to barcode SBW25. This process was repeated 25 times until a mutant library of sufficient diversity was reached. Following the construction of the backbone vector, plasmids with 25N random sequences were used to PCR amplify the vector, the product was ligated into a circular vector and amplified by transformation into *E. coli* Top10 cells (transformants were selected on agar plates supplemented with tetracycline), and a pool of plasmids were extracted from the resultant colonies and transformed into SBW25 with a pUX-BF13 helper vector (which expresses transposition machinery but does not replicate in SBW25). Transformants were grown on selective agar plates, and the resulting colonies were harvested and pooled into a single library.

Panel C illustrates the 25 bp of a random barcode and an example of 5 bp of the conserved areas left and right of the barcode coming from the TN7 plasmid. In addition, panel C shows the position downstream of *glmS* where the barcoded vector integrates into the SBW25 genome and final orientation of the vector.

The vector has three key elements: (1) an origin of replication (pBR322 origin) that allows propagation in an *E. coli* host (the pBR322 origin does not allow replication in non-*E. coli* hosts); (2) a selectable marker (in our case, a gene-encoding tetracycline resistance: *tetA*) (3) paired Tn*7* integrative elements (L and R elements) between which the selectable marker and later the barcodes are situated. Elements 1 and 2 may require exchange with suitable alternative elements to enable the use of this vector in other model species. Transcriptional terminators were included on either side of the selectable marker to limit excessive expression of the antibiotic resistance gene, the downstream barcode (unintended transcription of which may cause deleterious peptides), or the neighbour genes at the integration site.

Integration of random sequences (the barcodes) requires several rounds of cloning before the final barcoded library is delivered to the desired target species. Transformation also needs to be performed multiple times to achieve high diversity of the barcoded library. During this process—detailed in supplementary files, visually summarised in (Fig. 1, Panel B), and recounted in the following paragraphs—care should be taken to ensure the frequency of each barcoded genotype is as even as possible. Unevenness implies one or several barcodes are at a high frequency, which increases the likelihood that spontaneous mutations will occur in the lineage of those genotypes, reducing the resolution at which beneficial mutants can be quantified. To this end, repeated restriction digestion was used to limit ancestral vector sequences and the number of transformants was carefully monitored to prevent an overrepresentation of barcodes from any single transformation.

To integrate random sequences into the previously described vector, the backbone was amplified with primers (see Table S3) containing randomly generated 25 bp sequences (the “barcodes”) using a fast-cloning approach (Li et al. 2011). The PCR product was digested with DpnI, which specifically targets multiple methylated restriction sites found in the non-amplified backbone plasmid. The resulting products were transformed into electrocompetent TOP10 cells to ligate the linear PCR product into a circular plasmid and amplify ligated plasmids. Transformed colonies from one plate were counted to estimate the total number of transformants (with an average of ~ 23,000 CFU per transformation). The remaining colonies were harvested, resuspended in LB and plasmids extracted over several miniprep columns. The protocol was repeated 25 times to improve barcode diversity. An estimated 580,000 transformant colonies were harvested for plasmid extraction from all 25 transformations. The plasmids underwent an enzymatic digestion step to limit the occurrence of the ancestral non-barcoded plasmid to < 0.001% in the final barcoded population (note that without the final digestion, the ancestral barcode-free sequence was measured at about 1% of the final population of transformed SBW25).

Clonal populations of SBW25 were prepared for transformation with the barcoded vector using pre-established methods (Choi and Schweizer 2006). The barcoded plasmid and the pUX-BF13 helper plasmid (Bao et al. 1991) were transformed into SBW25 by electroporation. Cells from each transformation were plated on selective plates with a dilution series to estimate the total number of transformants. Importantly, the total number of harvested SBW25 transformants was always less (~ 25%) than the number of *E coli* transformants from which plasmids were extracted. Limiting the number of SBW25 transformants reduces the potential for transformation with the same barcoded vector, thus, increasing diversity of the final library. The estimated number of unique barcodes was ~ 100,000. To further increase library diversity, the library-making process was repeated 25 times drawing upon plasmids from independent *E. coli* transformations. Because plates with more colonies have smaller colonies, measures of OD were made of resuspended cells with adjustments to dilutions to ensure equal contributions of each transformation to the final library of cells. Each of the 25 transformations was mixed in a final volume of ~ 50 mL, which was then vortexed thoroughly and frozen for later experiments.

Following library construction, simple quality control checks are necessary. The most important check involves the determination of the fraction of clones that do not contain barcodes. Such unwanted cells—those containing no Tn*7* and those with Tn*7* but no barcode—can appear in the final library and arise as, for example, a consequence of imperfect antibiotic counter-selection or incomplete restriction digestion of the original plasmid. This fraction, if large, would mean that numerous mutational events are unassociated with a unique sequence and, thus, go undetected. This check was made by sequencing across the Tn*7* insertion site in 96 independent clones. The resultant data showed that all 96 contained unique barcodes [see (Theodosiou et al. 2023)].

### Library Preparation

Identification and quantification of barcoded lineages in populations require amplicon sequencing. Our approach involved a two-step strategy for amplification: (1) amplification via PCR of the barcoded region (using primers specific to the barcoded region) and (2) amplification with Illumina-specific primers (this product was then sequenced on Illumina platforms, see Supplementary material: Amplicon-library preparation protocol). Table S4 and S5 list primers used for the rounds of PCR. The first pair of primers features sequencing primer-binding sites, 8N randomers to detect amplification biases (these are optional), a smaller heterospacer (see below), and the barcode vector-specific sequence for annealing of the primers. The heterospacer consists of either none, one, or two extra nucleotides, with all three primers mixed to reduce the saturation of the flow cell with signals from the same sequenced nucleotide. The second primer pair consists of the P5 or P7 flow cell binding regions, eight nucleotide indices to allow identification of the reads, and finally, a region complementary to the first set of primers to allow amplification of the initial PCR product. Together, this results in a ~ 310 bp product, with the barcoded region able to be sequenced from either end using 150 bp paired-end reactions.

At the point of PCR, it is necessary to be aware of problems arising from the amplification of random barcode sequences—a problem that becomes more acute with each amplification cycle. It was observed during the design of the library-generation protocol that more than 18 total PCR cycles of the barcoded region resulted in multiple large aberrant amplicons (see Figure S1). This led to the design of a limited cycle approach—with the added benefit of reducing potential PCR jackpots—which involved extracting large quantities of DNA from the experimental samples (~ 1 μg). This means that many cells in the population are sampled and ultimately provide greater resolution on the frequency of each barcode. After testing several commercial kits, a chloroform-based DNA extraction method was employed because this maximised extraction of DNA from cell pellets (see Supplementary material: Salt DNA extraction protocol).

The DNA extracted from colonies was used as a template for the first round of PCR of 13 cycles using a high-fidelity DNA polymerase. This first reaction was performed in quadruplicate per biological sample. The four PCR products were then purified into a single concentrated sample, which was used as a template for the second round of PCR (of only 5 cycles). The final amplicon was then purified of remaining primers and primer dimers with a magnet-based fragment size selection kit. The purified product was then quality controlled with electrophoresis (see Figure S2) and amplicon sequenced using an Illumina MiSeq DNA Flex Library Prep Kit.

### Read Processing and Library Assessment

Following construction of a barcode library, the size and diversity of the library need assessment. This section describes a pipeline for extracting barcodes from sequencing data and then suggests an experimental protocol for testing the reproducibility and quality of the barcoded library.

When acquiring sequence data, it is advisable to check the quality of the amplicon sequences by filtering low-quality reads to avoid erroneous results in downstream analysis. If paired-end data are acquired, it is suggested to merge the reads, retaining the highest quality reads. The user should check that barcode sequences have similar length (± 2 bp) to the anticipated length (following the barcode design see Library Creation section), and only barcodes close to the expected values should be used. Moreover, it is best practice to map amplicon sequences to a reference sequence, ensuring that the amplicon sequences are in the same direction (sequences might be reversed or be in the reverse complement state after merging). Finally, barcode sequences should be retrieved by extracting sequence data that map within the coordinates of the reference barcode sequence. Alternatively, barcodes can be extracted based on the conserved areas left and right of the barcode (Fig. 1, Panel C) as in the *bartender* pipeline (Zhao et al. 2018). Using this approach, all sequences must have the same direction as the reference.

To assess the quality and reproducibility of the barcoded SBW25 library, three independent overnight cultures were initiated from the frozen stock (inoculated with ~ 1% V/V). Genomic DNA was extracted from all three populations, resulting in three amplicon libraries for sequencing.

After paired-end sequencing with MiSeq, we obtained two fastq files for each sample, each containing approximately 1.5 million sequences. The length of each sequence was around 150 bp, which included the barcoded area (~ 25 bp) and a conserved area left and right of the barcode (~ 120 bp). To ensure read quality, we used fastQC version 0.11.8. We then merged the paired-end reads using PEAR version 0.9.8 (default options), which keeps the read with the highest overall PHRED score (Zhang et al. 2014). To ensure that all sequences were in the same direction and orientation, we mapped all sequences to a reference barcode sequence using Minimap2 version 2.24 (Li 2018) with default options, requiring a minimum mapping quality of 30. Finally, we extracted the sequence reads that mapped to the reference barcode using a custom pipeline (see Data availability section).

**Fig. 2:**
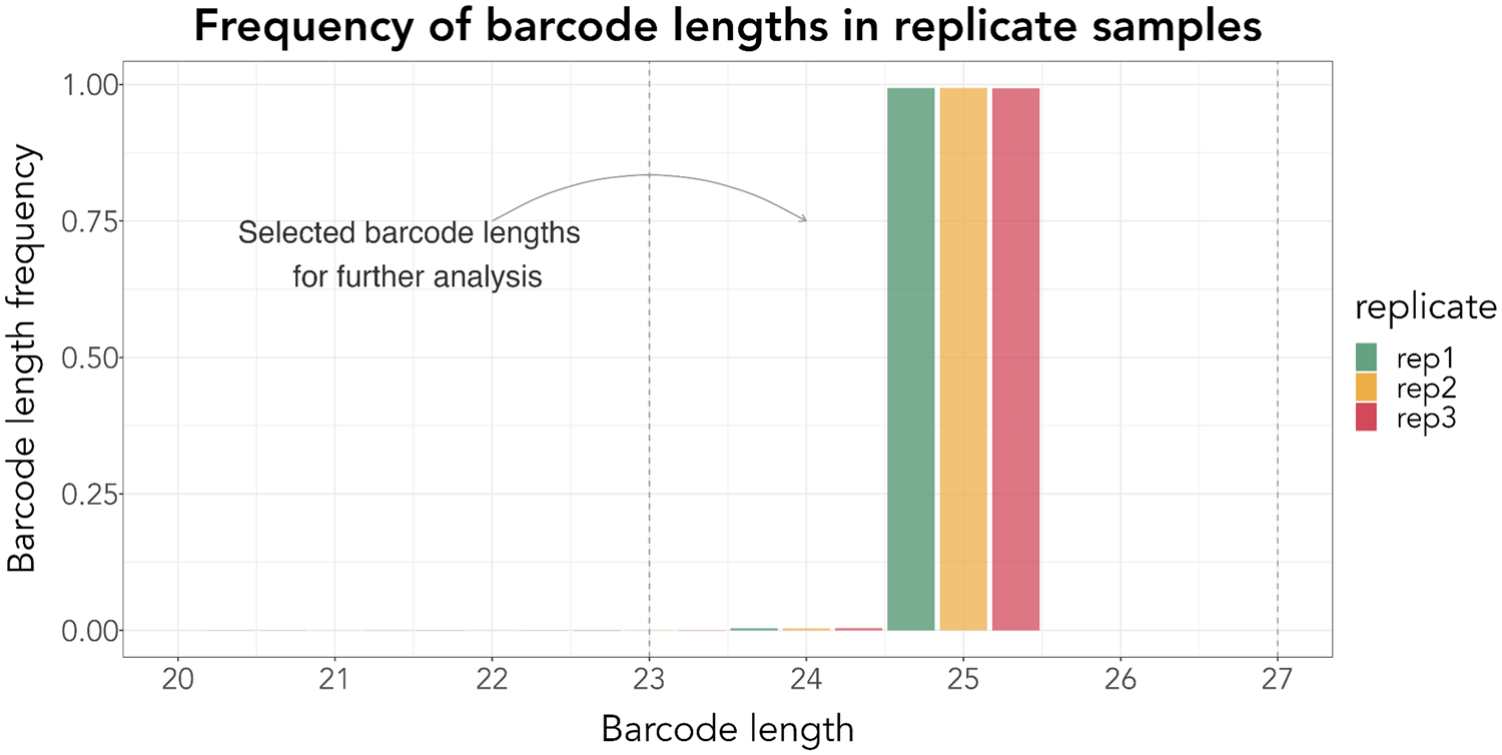
The frequency of the length of all barcodes was estimated for each replicate. In all replicates over 99% of all barcodes had an anticipated length of 25 bp. The plot shows the frequency of barcode lengths in each replicate, with different colours indicating each replicate. The x-axis indicates the barcode length, and the y-axis shows the frequency of each barcode length. The dotted lines represent the barcode length threshold, 23–27 bp, we used to select barcodes for further analysis

**Fig. 3:**
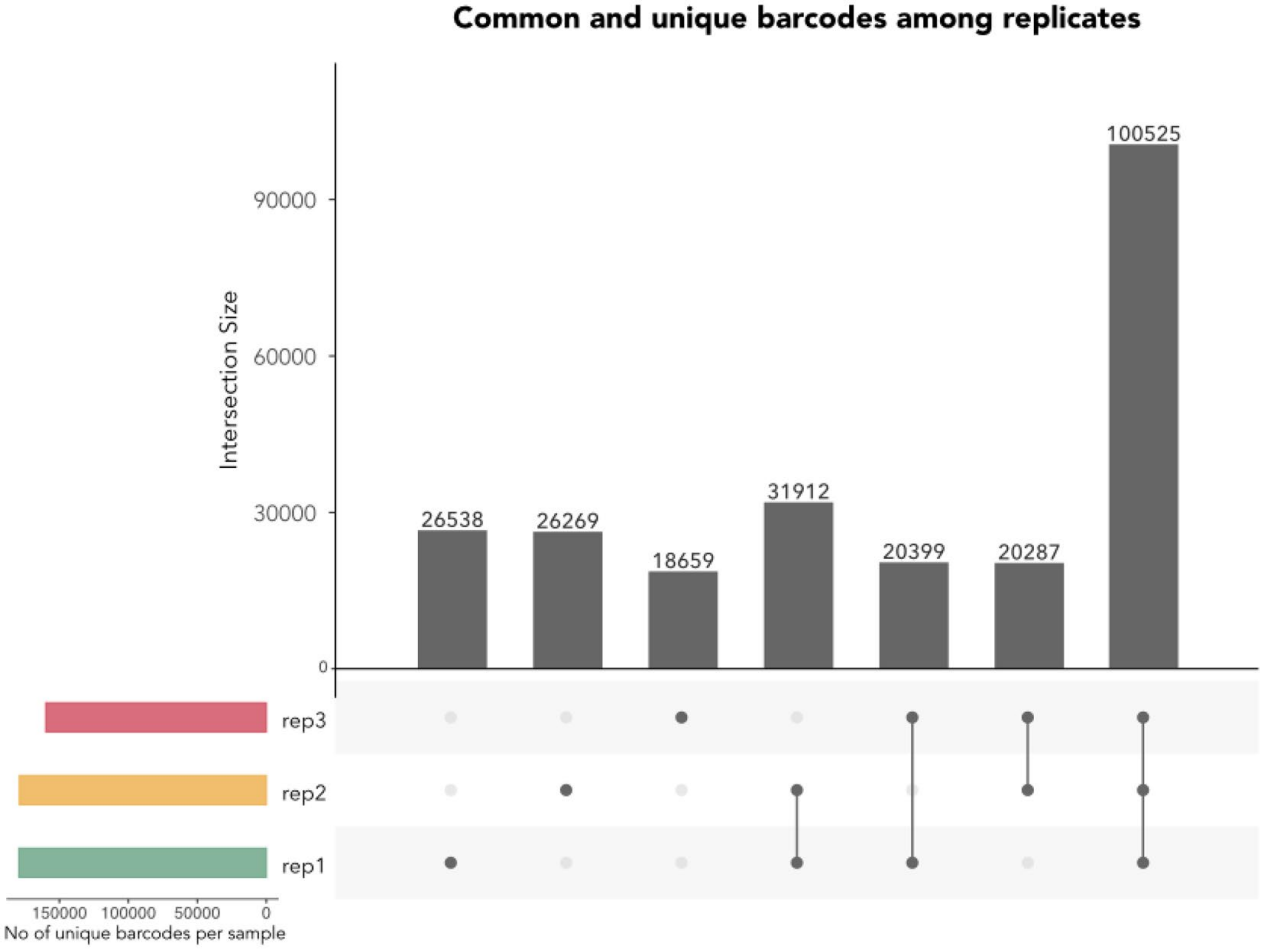
Common and unique barcodes among replicates. The UpSet plot shows the common and unique barcodes after comparing all sets of replicates. The coloured bar plot at the lower-left part of the UpSet plot shows the number of unique raw barcode sequences. The dot matrix indicates the raw barcode reads in each set of replicates. ~ 55% of all barcodes are common in all three replicates

**Fig. 4:**
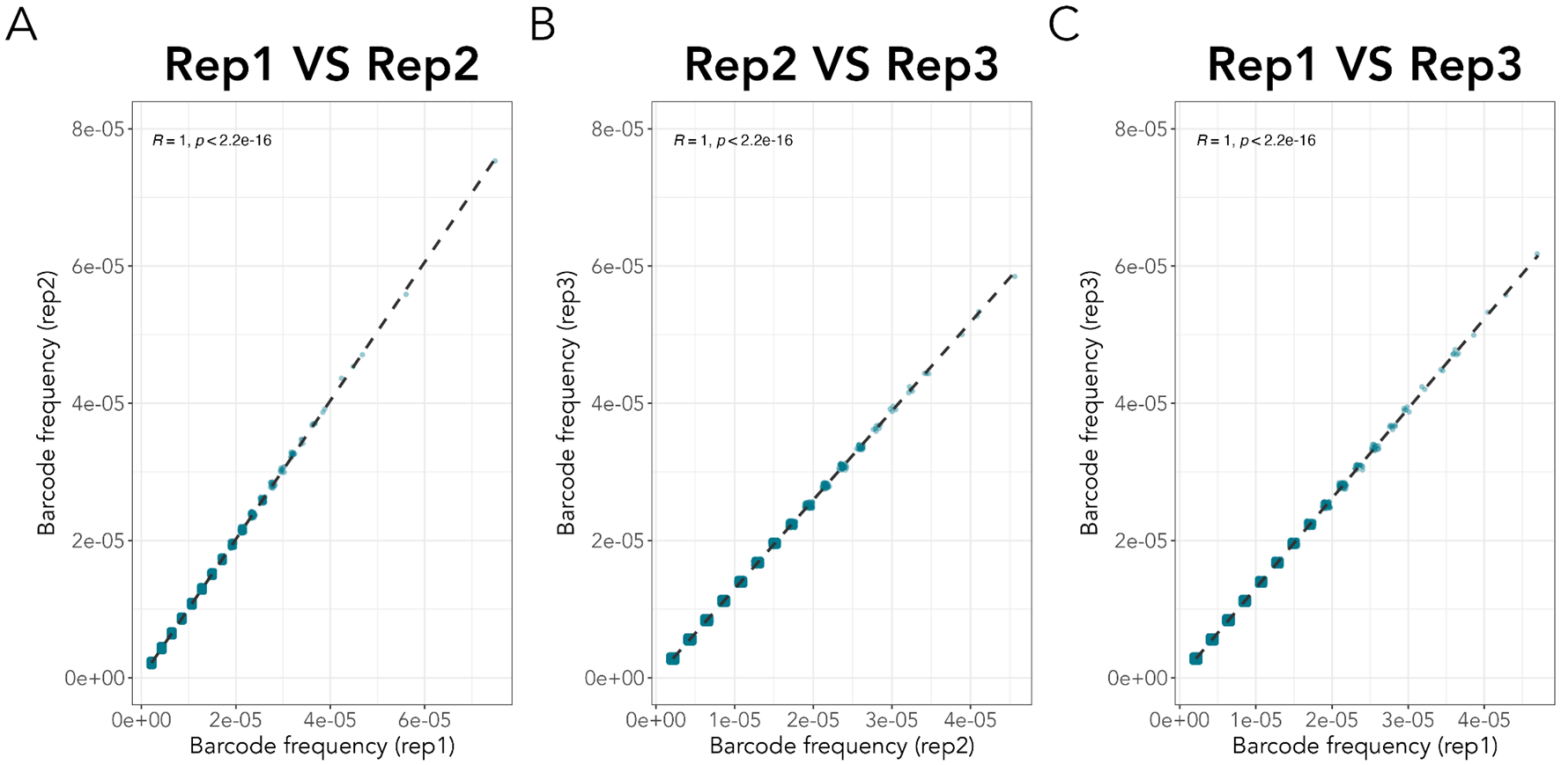
Pairwise comparisons of the barcode frequency between replicates. Scatterplots show the comparison of barcode frequencies between replicates using Pearson correlation. Panels A, B, and C compare replicates 1 and 2, 2 and 3, and 3 and 4, respectively. Each panel displays the R coefficient and *p* value at the top, with an R coefficient of 1 indicating a high similarity of the barcode frequencies across all replicates. To improve visual clarity, all points in the graph are slightly shifted using the ‘geom_jitter’ function from ggplot2 v.3.4.0, with a width and height of 0.0000005

Sequence mapping revealed 179.374 unique barcoded sequences from replicate 1178.993 from replicate 2 and 159.870 from replicate 3 (see Table S6). More than 98% of all barcodes in all replicates had the expected length of 25 ± 2 bp (Fig. 2) and could be used for downstream analysis. A pairwise comparison for all replicates was made to find common barcodes (Fig. 3), and frequencies were compared using Pearson correlations (Fig. 4). Barcode frequencies between replicates were highly similar, leading to an *R* = 1 in all cases, indicating high reproducibility. Across replicates, no specific barcodes were overrepresented, and barcodes were present at low frequencies, making our barcoded library suitable for experimental evolution (Figure S3). Around 56% of the unique barcodes were found in all three replicates, although this can increase significantly if the barcodes are clustered based on their sequence similarity (the clustering of barcodes is not considered further in this article). Differences in the number of unique barcodes in each replicate can be attributed to two possible factors: (1) library preparation and the sequencing process are prone to error, leading to changes in the nucleotide sequence and, thus, to different unique barcode sequences; (2) Sampling effects: at each stage of culturing, sampling and amplicon sequencing, multiple bottlenecks exist that may alter final barcode diversity.

## Next Stages

Having prepared and evaluated libraries, attention turns to experiments where barcoding can be applied. A standard experiment is to set up replicates of genetically homogeneous barcoded populations in different ecological conditions (e.g. with and without predators, antibiotics, salinity, or fluctuating environments), sample populations daily, amplify the barcoded region from the population, and sequence the amplicons. The resulting sequences allow changes in the frequencies of lineages to be tracked. Aided by mathematical models, it is possible to infer the number of beneficial mutations, their fitness effects, and how these affect the population dynamics of lineages. It is of interest to quantify competition among lineages and the contribution of ecology and evolution to lineage population dynamics.

Beyond the analysis of adaptive evolution in single populations, barcoded organisms can be used to explore a range of questions in ecology and evolution. For example, introducing a barcoded population to a community of microbial species allows analysis of the eco-evolutionary dynamics of the focal type embedded in a community [referred to as an ESENCE approach (Lenski 2017)]. To do so, a population of the model or nonmodel organism can be barcoded and then introduced into experimental cultures composed of complex microbial communities. The dynamics of that focal strain can then be observed over time. Using this procedure, the effects of manipulation of the community (such as specific composition, diversity, resource use, or adaptation to resources etc.) can be assessed.

A second line of inquiry concerns ecological factors shaping diversification in the face of dispersal and recolonisation events. Populations of barcoded cells can be subjected to different regimes of dispersal or disturbance over short time scales.

Once experimental data have been acquired, further challenges remain, such as clustering of sequencing data and inference of eco-evolutionary dynamics. The current methodology (Levy et al. 2015; Ba et al. 2019; Kinsler et al. 2020; Vasquez et al. 2021; Avecilla et al. 2022; Johnson et al. 2023; Li et al. 2023) for analysing barcoding data has been tailored to specific types of experiments that assume absence of ecological processes, such as frequency-dependent selection. There is a considerable need to build pipelines for analysis that encompasses more complex and realistic eco-evolutionary scenarios. The challenges presented require extensive development and description and will be the subject of a forthcoming paper.

## Conclusions

In this article, we have provided a step-by-step guide for barcoding microbial cells: from library preparation to amplicon sequencing, including methods to assess sequence data quality. As an example, a protocol to barcode *Pseudomonas fluorescens* SBW25 has been provided, and procedures have been emphasised, which enable maximum diversity of the resulting library. Barcoding SBW25, or any other strain, constitutes a powerful and versatile system for tracking the eco-evolutionary dynamics of populations—in a multitude of contexts—through time. We hope that the protocols described, along with the accompanying discussion, serve to promote the construction of barcoded libraries in a diverse array of organisms and the development of new experimental possibilities.

## Supplementary Information

Below is the link to the electronic supplementary material, which includes supplementary figures and tables.

Supplementary file1 (DOCX 3432 KB)

## Data Availability

All data is deposited in the following zenodo repository 10.5281/zenodo.7703993. Code and a tutorial for analysing the data are deposited in the following publicly available GitLab repository https://gitlab.gwdg.de/loukas.theodosiou/sbw25-barcoding.
