# Supplementary material for: Barcoding Populations of Pseudomonas fluorescens SBW25
Source: J Mol Evol. 2023 Apr 25;91(3):254–62. doi: 10.1007/s00239-023-10103-6 (PMC10275814; doi:10.1007/s00239-023-10103-6)
Supplement: Supplementary file 1 — Supplementary file1 (DOCX 3432 KB) [file 239_2023_10103_MOESM1_ESM.docx]

**Supplementary Material**

1. **Protocol for chromosomal integration of barcodes**

Protocol to generate barcoded *Pseudomonas fluorescens* SBW25. This protocol starts from a specifically generated cloning vector (see MPB_15151_plasmid_unbarcoded in (Theodosiou et al. 2023) and details the introduction of the barcoded region and subsequent cloning of intermediate *E coli* hosts and the final SBW25 host. This process was repeated 25 times until sufficient diversity of the resulting library was reached (i.e. a SBW25 library of >100,000 barcodes).

1. The plasmid of MPB15151 (is extracted using the Miniprep Kit (Qiagen). The plasmid is digested with KpnI-HF (NEB) to linearise it following manufacturers' instructions and purified using a PCR purification kit (Qiagen).
2. Using Primers MPB15151_BCSDM_25r4N_F and MPB15151_BCSDM_17HRF_R (see Table S3 for more details), 50 ng of the digested plasmid was amplified using primers encoding the barcoded sequence in 20 μL volumes using ‘Q5 high-fidelity DNA polymerase’ (NEB) following the manufacturer's instructions.
3. In order to limit unmodified MPB15151 from subsequent cloning steps, the entire PCR product was digested for 60 mins with the restriction enzyme DpnI (NEB) (following manufacturers' instructions) and purified again with the PCR purification kit (Qiagen). DpnI digestion helps limit transformation with the ancestral plasmid.
4. 100 ng (approx. 1 μL) of the digested amplicons were then transformed into ‘One Shot TOP10 Electrocomp *E. coli* (Invitrogen) using a ‘Micropulser’ electroporator (Bio-Rad), using a 0.1 cm electrode gapped ‘Gene Pulser Cuvette’ (Bio-Rad). Cells were immediately revived with 500 μL SOC media in 1.5 mL microcentrifuge tubes, incubated at 37 °C and orbitally shaken at 1000 rpm.
5. The aliquot of revived cells was plated across 8 agar plates supplemented with 10 ug/mL tetracycline, with one extra plating at a lower volume of cells (5 μL) to derive an estimate of transformants. Assuming 25 transformations, the researcher should prepare sufficient plates ahead of time (~230 agar plates in our case). Plates were incubated for 48 hrs at 37 °C.
6. Resulting colonies from each transformation were harvested by scraping directly off the plate and resuspended in 5 mL of LB supplemented with tetracycline (10 μg/mL)
7. 1 mL from each resuspension was cryogenically stored, and the remaining 4 mL was plasmid extracted using miniprep (Qiagen) - using 1 column per mL of cells - with columns eluted with 30 μL of EB warmed to 60 °C. Note at this stage, the plasmids are in their fully barcoded form (see the sequence of “MPB15151_barcoded_25N” (Theodosiou et al. 2023)).
8. Extracts from each miniprep were mixed, and 4 μg of plasmid were digested with KpnI-HF in 50 μL volumes. This step limited the remaining ancestral vectors which do not contain the barcoded sequence (the barcode effectively replaces the KpnI restriction site). The KpnI-HF digested plasmid was purified using a PCR purification kit (NEB).
9. Electrocompetent *Pseudomonas fluorescens* SBW25 was prepared for electroporation using standard procedures (Choi and Schweizer 2006). To do so, overnight cultures were initiated from isogenic cryogenic stocks and grown with shaking over ~18 hrs at 28 °C. For each transformation, 1 mL of overnight culture was washed over five cycles of centrifugation and washing with room temperature 300mM sucrose with the last resuspension made in 30 μL of sucrose solution. Cells were kept at room temperature until electroporation a few minutes later.
10. Electrocompetent cells were then co-transformed with 150 ng of barcoded vector and the pUX-BF13 helper plasmid (Bao et al. 1991) using the same methods of electroporation and revival as the *E. coli* electroporation (see above). Cells from each transformation were plated on 5 LB agar plates (supplemented with 10 μg/mL tet) with an extra plating with diluted cells to estimate the number of transformants. Plates were incubated for 48 hrs at 28 °C.
11. Colonies were scraped together, resuspended in 3 mL of KB media, and vortexed for 40 secs. Colonies from the plate of diluted cells were counted to estimate the number of transformants harvested, so the final diversity of the resulting library can be estimated. Because plates with more colonies would have smaller colonies, measures of OD were taken of the resuspensions, and mild dilutions adjusted the volume of reach resuspension with KB to ensure equal contributions of each transformation to the final library of cells. As this step was performed on the same day, each of the 25 transformations was mixed in a final volume of ~50 mL, which was then vortexed thoroughly and frozen over approximately 50 aliquots with glycerol saline for later experiments.

**2. Protocol for amplicon-library preparation**

Protocol to generate indexed amplicons for 150 bp paired-end sequencing.​ Note: do not increase the number of cycles, which may cause aberrant-sized amplicons (see Figure S1). A final amplicon product of ~30 ng/μL is sufficient for Illumina sequencing.

1. Obtain high-concentration genomic DNA (approximately 500 ng is used in this protocol for each PCR reaction).
2. Perform 1st PCR (Table S1). Perform 4 PCR reactions (40 μL each) using DNA from the same sample. Use 500 ng of DNA as a template in each 40 μL reaction. Add the Q5 polymerase last. The three primers composing Ampseq_HS0/1/2_F+24 and Ampseq_HS0/1/2_R-63 are mixed in equimolar ratios. Expect a final product of ~241 (The primers are ~64 and ~65 nucleotides long, and the template in between is 112 bp; for details, see Table S1, and for the primer sequences, see Table S4)
3. Gel electrophoresis is confirmed by amplicon (not a must at this point).
4. Remove genomic DNA and primers by DNA purification. Purify with Qiagen PCR purification kit - the main alteration is that the four PCR products are passed through one column when initially loaded. Elute each column with 40 μL prewarmed H20. This is the template for the second PCR. A yield of about 5-50 ng/μL is expected at this stage.
5. Perform second PCR (Table S2) to add the second primer pair (see Table S5), which features the flow cell binding region and indices. A greater concentration of primer is used than typical in order to prevent template dimerisation. Expect a ~310 bp product (the second primers are 37 and 32 nucleotides long, and the template in between is 241 nucleotides).
6. Purify with Size selection to remove products <150 bp (i.e. the primers must be removed) using a ‘ProNex Size-selective purification System’ (Promega) or similar size-selective strategy.
7. Run standard quality control methods before sequencing (check amplicon size ideally by automated electrophoresis to ensure the correct sized amplicon. Establish DNA concentration by fluorometry.

**Table S1:** PCR protocol for the 1st PCR of the library preparation protocol. Panel A indicates the quantity of the PCR reagents, and Panel B indicates the PCR cycle conditions.

**Panel A: PCR protocol**

| Product | Quantity |
| --- | --- |
| Q5 2x    Ampseq_HS0/1/2_F+24 (10uM)    Ampseq_HS0/1/2_R-63 (10uM)    DNA  ddH20 | 20 μl    2.0 μl    2.0 μl    500ng (~1 μL)  to 40 μL total |

**Panel B: PCR cycle conditions**

| Temperature | Time |
| --- | --- |
| 98 °C    98 °C    64 °C    72 °C  72 °C    4 **°**C | 30 sec    10 sec    20 sec    20 sec x 13 passes  1 minute  ∞ |

**Table S2:** PCR protocol for the 2nd PCR of the library preparation protocol. Panel A indicates the quantity of the PCR reagents, and Panel B indicates the PCR cycle conditions.

**Panel A: PCR protocol**

| Product | Quantity |
| --- | --- |
| Q5 2x    Adf_F-X (10μM)    Adf_R-X (10μM)    1st PCR product | 25 μl    7.5 μl    7.5 μl    10.0 μL |

**Panel B: PCR cycle conditions**

| Temperature | Time |
| --- | --- |
| 98 °C    98 °C    68 °C    72 °C  No extension | 30 sec    10 sec    20 sec    30 sec x 5 passes |

**3. Protocol for DNA extraction of bacterial cultures**

1. Collect 1.8 mL of a dense (~ 2 x 10^9^ cells/mL) overnight bacterial culture in a 2 mL eppendorf tube, centrifuge at 14500xg for 15 min and discard the supernatant.
2. Add 500 µL HOM-Buffer (HOM-Buffer = 80 mM EDTA, 100 mM Tris, 0,5 % SDS) and 5 µL RNase.

Note: We used the RNase A Solution 4 mg/mL from Promega

1. Incubate samples for 1 hr, at 37 °C in an orbital tube shaker at 400rpm.
2. Add 5 µL Proteinase K [0.20 mg/mL].

Note: We prepared beforehand aliquots of Proteinase K from the stock solution *QIAGEN Proteinase K ready-to-use solution* [20 mg/mL] with final concentration of [0.20 mg/mL].

1. In a tube shaker, incubate samples at 55 °C overnight (500 rpm).
2. Add 500 µL of Sodium-Chloride solution (4.5 M) and incubate samples for 10 min at 4 °C.
3. Add 300 µl Chloroform and mix gently for 15 min in a rotating mixer.

Note: Chemicals such as chloroform should be used carefully under a fume hood.

1. Centrifuge for 10 min at 10000 rpm.
2. Transfer the upper phase (ca. 850 µL) into a new centrifuge tube.
3. Add 595 µL of 100 % isopropanol and mix for 5 min in a rotating mixer.
4. Centrifuge for 10 min at 13000 rpm and discard supernatant.
5. Add 500 µL 70 % Ethanol (dilution from 100 % ethanol for molecular biology) and incubate for 5 min at room temperature.

Note: We used Ethanol absolute Molecular biology grade from AppliChem

1. Centrifuge for 10 min at 13000 rpm and discard the supernatant.
2. Repeat the last step and remove any residual supernatant.
3. Dry the pellet at room temperature.
4. Add 30 µL of elution buffer and elute for 30 min at room temperature.
5. Place the samples overnight at 4 °C.
6. Measure DNA concentration using Qubit and protein/RNA contamination with Nanodrop.
7. Store samples at -20 °C until amplicon-library preparation.

**4. Primers used in this study**

All the primers that have been used for the barcoded library of SBW25 were HPLC purified, except for the initial fast-cloning primer encoding the random barcodes, which was PAGE purified. All primers are written 5’ to 3’.

**Table S3:** Primers that have been used for fast cloning the 25N randomer into the backbone plasmid

| Primer name | Primer Sequence | Primer Description |
| --- | --- | --- |
| MPB15151_BCSDM_25r4N_F | CGCGATGTCCACGAAGCTCTCCTACGNNNNNNNNNNNNNNNNNNNNNNNNNCAGTCCAGCGCCAACCAGATAAGTGAAATCTAGTTCC | Forward primer featuring a 25N randomer for fast cloning of the barcoding region into the backbone plasmid (MPB15151) |
| MPB15151_BCSDM_17HRF_R | GAGCTTCGTGGACATCGGCCCAAGCTTCTCGAGGAATTC | Reverse primer for fast cloning of the barcoding region into the backbone plasmid |

**Table S4**: A list of the primer sequences were used for the 1st PCR of the library preparation for sequencing. The green colour at the 5’ site indicates an Illumina sequencing primer binding region, the *8N* a random sequence, and the red colour is a heterospacer. Lastly, the non-coloured area at the 3’ site indicates the *in-situ* annealing sequence.

| Primer name | Primer Sequence |
| --- | --- |
| Forward primers |  |
| Ampseq_HS0_F+24    Ampseq_HS1_F+24    Ampseq_HS2_F+24 | GTGACTGGAGTTCAGACGTGTGCTCTTCCGATCTNNNNNNNNAATTCCTCGAGAAGCTTGGGC  GTGACTGGAGTTCAGACGTGTGCTCTTCCGATCTNNNNNNNNTAATTCCTCGAGAAGCTTGGGC  GTGACTGGAGTTCAGACGTGTGCTCTTCCGATCTNNNNNNNNGTAATTCCTCGAGAAGCTTGGGC |
| Reverse primers |  |
| Ampseq_HS0_R-63      Ampseq_HS1_R-63      Ampseq_HS2_R-63 | ACACTCTTTCCCTACACGACGCTCTTCCGATCTNNNNNNNNGGTGTAGCGTCGTAAGCTAATAC  ACACTCTTTCCCTACACGACGCTCTTCCGATCTNNNNNNNNAGGTGTAGCGTCGTAAGCTAATAC  ACACTCTTTCCCTACACGACGCTCTTCCGATCTNNNNNNNNTCGGTGTAGCGTCGTAAGCTAATAC |

**Table S5:** A list of the primer sequences used for the 2nd PCR of the library preparation for sequencing. The red text at the 5’ site indicates the P5 or P7 flow cell binding regions, while the black text, *8N*, depicts a random sequence for indexing. Finally, the green text at the 3’ site indicates the annealing sequence for 1st PCR template.

| Primer name | Primer Sequence |
| --- | --- |
| Forward primers |  |
| Adf_F-1   Adf_F-2   Adf_F-3   Adf_F-4   Adf_F-5 | AATGATACGGCGACCACCGAGATCTACACAACCGCAT ACACTCTTTCCCTACACGACGC  AATGATACGGCGACCACCGAGATCTACACAAGGCCTT ACACTCTTTCCCTACACGACGC  AATGATACGGCGACCACCGAGATCTACACAGAGTGTG ACACTCTTTCCCTACACGACGC  AATGATACGGCGACCACCGAGATCTACACCACAAGTC ACACTCTTTCCCTACACGACGC  AATGATACGGCGACCACCGAGATCTACACCGTTCCTA ACACTCTTTCCCTACACGACGC |
| Reverse primers |  |
| Adf_R-A   Adf_R-B   Adf_R-C   Adf_R-D   Adf_R-E | CAAGCAGAAGACGGCATACGAGAT AACCGGAA GTGACTGGAGTTCAGACGTGTGC  AATGATACGGCGACCACCGAGATCTACAC AAGGCCTT ACACTCTTTCCCTACACGACGC  AATGATACGGCGACCACCGAGATCTACAC AGAGTGTG ACACTCTTTCCCTACACGACGC  CAAGCAGAAGACGGCATACGAGAT CGTTCGTT GTGACTGGAGTTCAGACGTGTGC  CAAGCAGAAGACGGCATACGAGAT CTGTTCAC GTGACTGGAGTTCAGACGTGTGC |

**Table S6:** Summary table indicating the total number of sequences, mapped sequences to reference barcode and unique barcode sequences for each replicate.

| Replicate | Total number of sequences | Mapped sequences to the reference | Unique barcodes |
| --- | --- | --- | --- |
| rep1 | 467846 | 467773 | 179374 |
| rep2 | 463383 | 463314 | 178993 |
| rep3 | 357691 | 357602 | 159870 |

**Supplementary Figures**

**
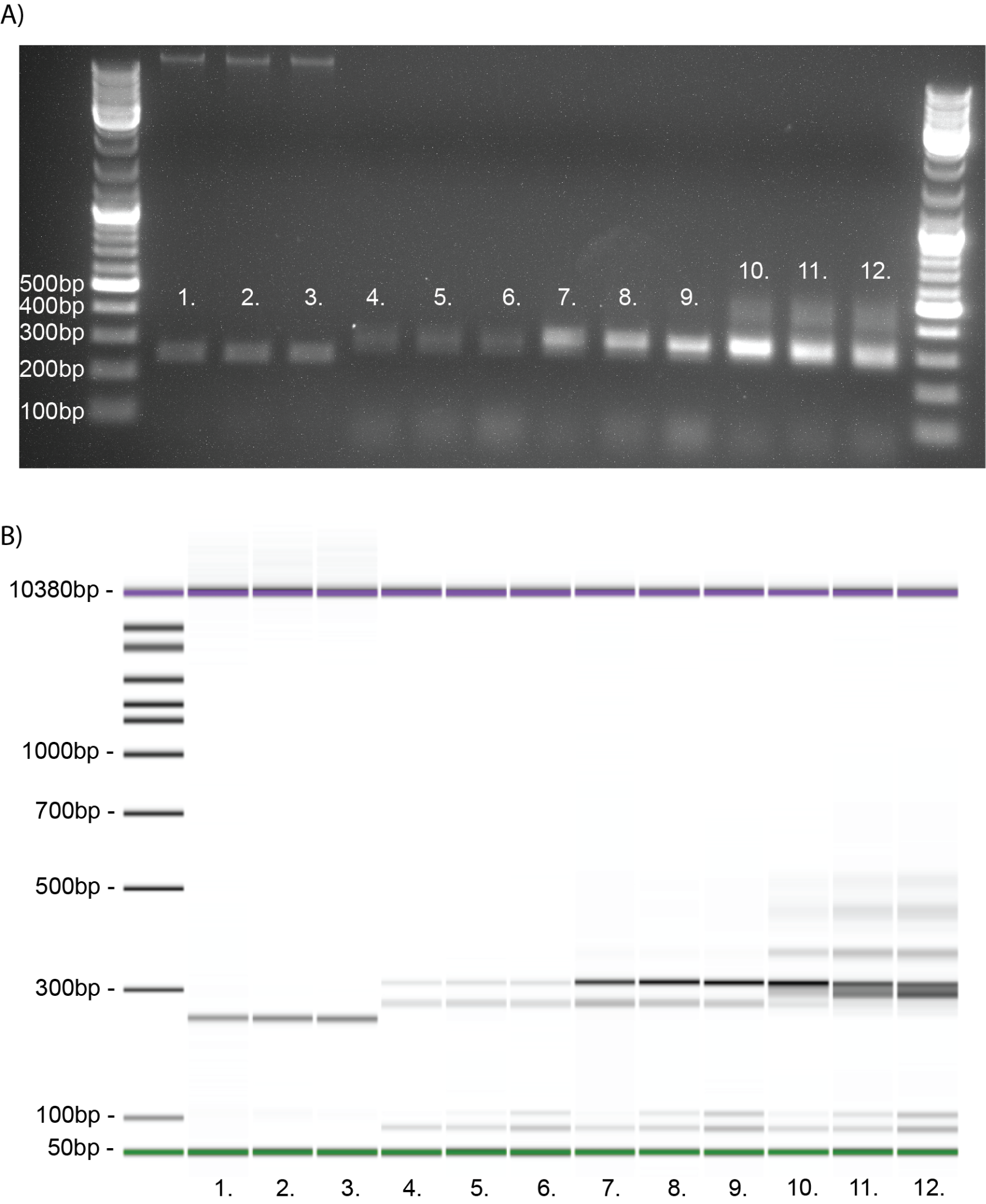
**

**Figure S1:** Excessive cycles of the second PCR causes aberrant amplicon sizes larger than ~313 bp.

Displayed are images of purified amplicon library products run on 1) agarose gel and 2) chip-based electrophoresis following amplification with internal and then external illumina-specific primers. The products run in lanes 1-3 resulted from the initial 13 cycles (run with primers Ampseq_HS0/1/2_F+24, and Ampseq_HS0_R-63, see Table S4). The products were purified and then subjected to either 2 (lanes 4-6), 4 (lanes 7-9) or 6 (lanes 10-12) cycles of secondary PCR with illumina primers (specifically Adf_F-1 and Adf_R-A, see Table S5). After 6 secondary PCR cycles, larger amplicons appeared that were greater than the expected size of ~313 bp. The PCR’s were performed as described in “Protocol for amplicon-library preparation” (see supplementary material), with the exception that initial primer concentrations were 0.5 μM for samples 4, 7 and 10 and 1.0 μM for samples 5, 8 and 11 to evaluate the impact of lower primer concentrations on the amplicons.

**
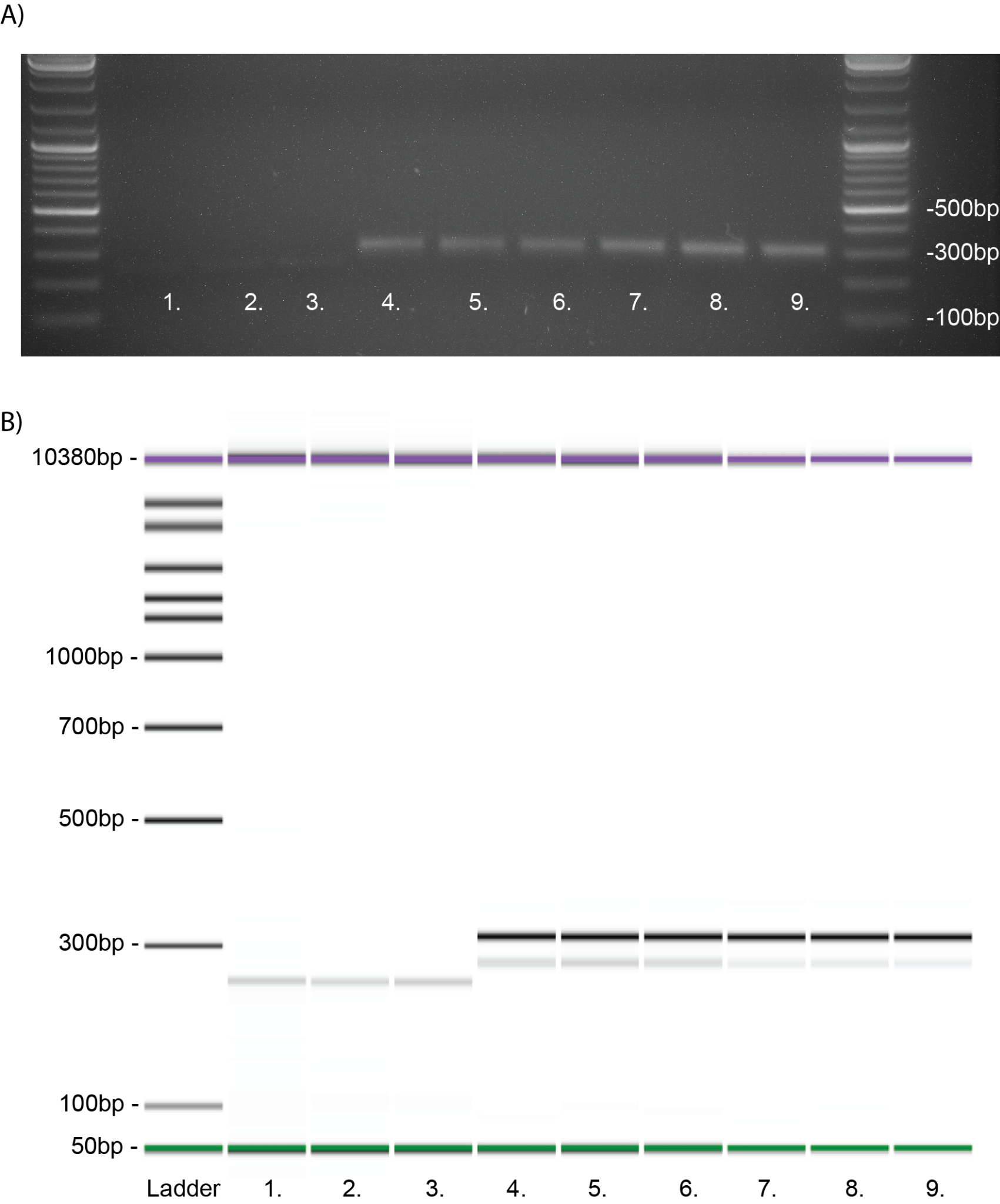
**

**Figure S2:** Appropriate size and appearance of second PCR amplicons as visualised by electrophoresis.

The images displayed show the correct appearance of amplicons of the barcoded region ready for sequencing as visualised through agarose gel (A) and chip-based electrophoresis (B). Lanes 1-3 represent purified products of the initial 13 cycles (with primers Ampseq_HS0/1/2_F+24, and Ampseq_HS0_R-63, see Table S4), resulting in barely visible products. These products were then purified and subjected to amplification with independent sets of illumina-specific primers (see Table S5) over 4 (lanes 4-6) or 5 cycles (lanes 7-9). It is important to note the faint lower band in lanes 4-9 in image B, which likely depicts primer-extension of a single strand of PCR products from the first 13 cycles. Based on the lack of other bands and the higher concentration of the intended band at ~313 bp, 5 cycles are recommended for the second PCR cycle. Additionally, it should be noted that the products depicted by lanes 7-8 are the same products later sequenced using MiSeq and described in Figures 2-4.

**
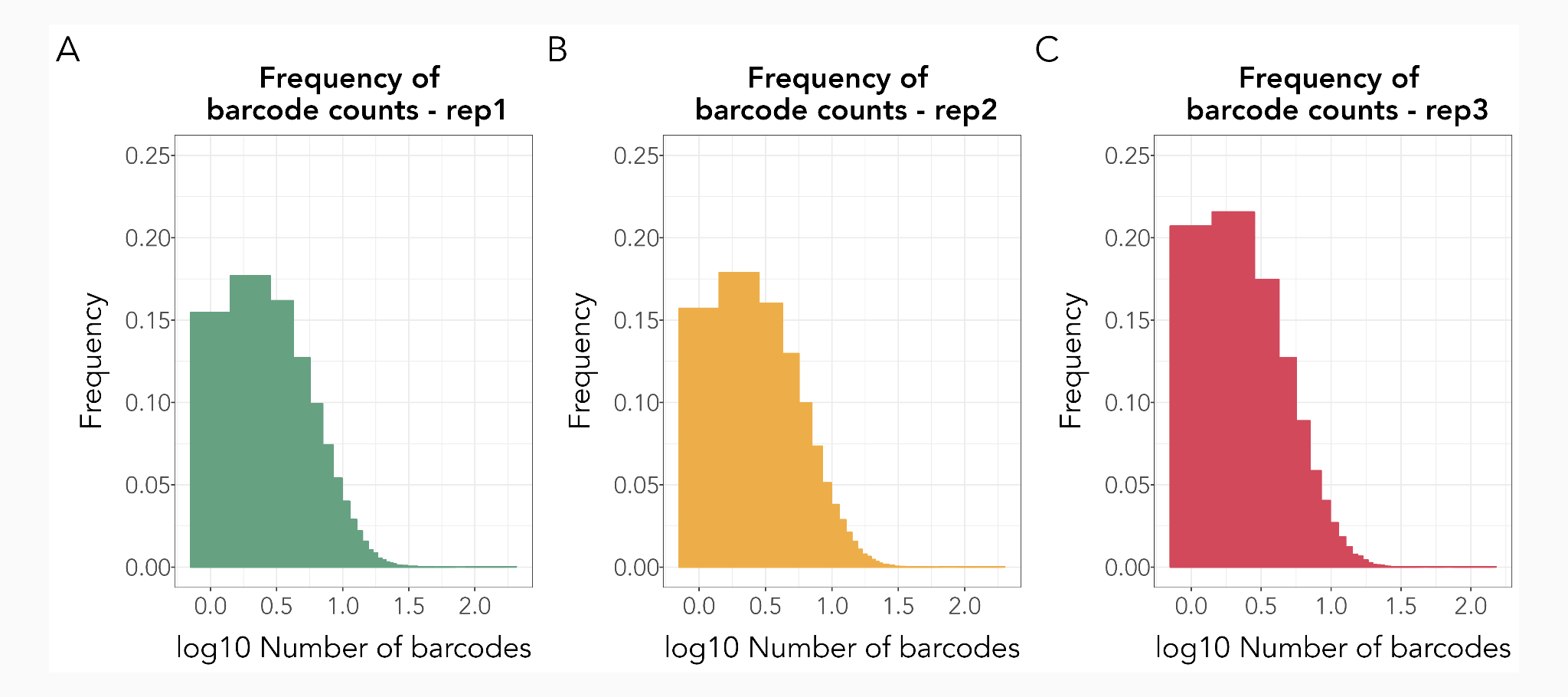
**

**Figure S3:** Distribution of barcode frequencies across replicates.

The figure presents frequency histograms of barcode counts for each replicate in a log10 scale. Panels A, B, and C correspond to replicate 1, 2, and 3, respectively. The histograms exhibit striking similarity across replicates, with most barcodes appearing at low frequencies. This observation indicates that the barcode pool is appropriate for experimental evolution without overrepresented barcodes.

**References**

Bao Y, Lies DP, Fu H, Roberts GP (1991) An improved Tn7-based system for the single-copy insertion of cloned genes into chromosomes of gram-negative bacteria. Gene 109:167–168. https://doi.org/10.1016/0378-1119(91)90604-A

Choi K-H, Schweizer HP (2006) mini-Tn7 insertion in bacteria with single *att*Tn7 sites: example *Pseudomonas aeruginosa*. Nat Protoc 1:153–161. https://doi.org/10.1038/nprot.2006.24

Theodosiou L, Farr AD, Rainey PB (2023) Barcoding populations of *Pseudomonas fluorescens* SBW25, Data repository, zenodo:10.5281/zenodo.7703993
